# Prenatal anaemia and risk of postpartum haemorrhage: a cohort analysis of data from the Predict-PPH study

**DOI:** 10.1186/s12889-024-18446-5

**Published:** 2024-04-12

**Authors:** Kehinde S. Okunade, Adebola A. Adejimi, Ayokunle M. Olumodeji, Atinuke Olowe, Olufemi A. Oyedeji, Iyabo Y. Ademuyiwa, Hameed Adelabu, Eselobu Toks-Omage, Austin C. Okoro, Nosimot Davies, Muisi A. Adenekan, Temitope Ojo, Kabiru A. Rabiu, Yusuf A. Oshodi, Aloy O. Ugwu, Festus O. Olowoselu, Olukayode O. Akinmola, Joseph A. Olamijulo, Ayodeji A. Oluwole

**Affiliations:** 1 Department of Obstetrics & Gynaecology, College of Medicine, University of Lagos, PMB 12003, Lagos, Nigeria; 2 Department of Obstetrics & Gynaecology, Lagos University Teaching Hospital, Lagos, Nigeria; 3 Centre for Clinical Trials, Research and Implementation Science, College of Medicine, University of Lagos, Surulere, Lagos Nigeria; 4 Department of Community Health & Primary Care, College of Medicine, University of Lagos, Surulere, Lagos Nigeria; 5 Department of Obstetrics & Gynaecology, Lagos State University Teaching Hospital, Ikeja, Lagos Nigeria; 6 Department of Nursing Science, College of Medicine, University of Lagos, Surulere, Lagos Nigeria; 7 Department of Haematology and Blood Transfusion, College of Medicine, University of Lagos, Lagos, Nigeria; 8 Faculty of Basic Medical Sciences, College of Medicine, University of Lagos, Surulere, Lagos Nigeria; 9 Department of Obstetrics & Gynaecology, Lagos Island Maternity Hospital, Lagos Island, Nigeria; 10 Department of Obstetrics & Gynaecology, Federal Medical Center, Ebute-Meta, Lagos, Nigeria; 11 Department of Obstetrics & Gynaecology, 68 Nigerian Army Reference Hospital, Yaba, Lagos Nigeria; 12 Department of Chemical Pathology, Lagos University Teaching Hospital, Surulere, Lagos Nigeria

**Keywords:** Blood loss, Lagos, Predict-PPH, Prenatal anaemia, WHO-defined PPH

## Abstract

**Background:**

Most previous clinical studies investigating the connection between prenatal anaemia and postpartum haemorrhage (PPH) have reported conflicting results.

**Objectives:**

We examined the association between maternal prenatal anaemia and the risk of PPH in a large cohort of healthy pregnant women in five health institutions in Lagos, Southwest Nigeria.

**Methods:**

This was a prospective cohort analysis of data from the *Predict-PPH* study that was conducted between January and June 2023. The study enrolled *n* = 1222 healthy pregnant women giving birth in five hospitals in Lagos, Nigeria. The study outcome, WHO-defined PPH, is postpartum blood loss of at least 500 milliliters. We used a multivariable logistic regression model with a backward stepwise conditional approach to examine the association between prenatal anaemia of increasing severity and PPH while adjusting for confounding factors.

**Results:**

Of the 1222 women recruited to the *Predict-PPH* study between January and June 2023, 1189 (97·3%) had complete outcome data. Up to 570 (46.6%) of the enrolled women had prenatal anaemia while 442 (37.2%) of those with complete follow-up data had WHO-defined PPH. After controlling for potential confounding factors, maternal prenatal anaemia was independently associated with PPH (adjusted odds ratio = 1.37, 95% confidence interval: 1.05–1.79). However, on the elimination of interaction effects of coexisting uterine fibroids and mode of delivery on this association, a sensitivity analysis yielded a lack of significant association between prenatal anaemia and PPH (adjusted odds ratio = 1.27, 95% confidence interval: 0.99–1.64). We also recorded no statistically significant difference in the median postpartum blood loss in women across the different categories of anaemia (*P* = 0.131).

**Conclusion:**

Our study revealed that prenatal anaemia was not significantly associated with PPH. These findings challenge the previously held belief of a suspected link between maternal anaemia and PPH. This unique evidence contrary to most previous studies suggests that other factors beyond prenatal anaemia may contribute more significantly to the occurrence of PPH. This highlights the importance of comprehensive assessment and consideration of various maternal health factors in predicting and preventing this life-threatening obstetric complication.

## Introduction

Postpartum haemorrhage (PPH) leads to approximately 80,000 annual deaths globally and is the primary cause of maternal mortality in many developing countries, including Nigeria [1]. It also increases the likelihood of adverse outcomes for newborns, such as preterm birth and low birth weight [2]. As per the World Health Organization (WHO), primary postpartum haemorrhage (PPH) is typically defined as the occurrence of blood loss surpassing 500 mL within the 24 h following childbirth [3]. A recent nationwide cross-sectional study of 38 maternal healthcare units in Nigeria public health facilities reported a PPH prevalence ranging from 0.4 to 16.8% [4]. Anaemia, typically defined as haemoglobin (Hb) concentration below 11.0 g/dL at any stage of pregnancy [5], ranks as the most prevalent medical disorder during pregnancy [6] and is also a major contributor to global maternal and perinatal morbidity and mortality [1]. It is a sign of inadequate nutrition and poor health [7]. About half of the women of reproductive age are anaemic in South Asia and Western and Central Africa, where anaemia is most common [2].

Anaemia plays a crucial role in contributing to maternal morbidity through molecular, cellular, and anaemia-induced hypoxia [8]. Mechanistic studies also suggest that preexisting anaemia might be associated with the incidence of PPH [9, 10] with possible mechanisms including increased blood flow from bleeding vessels due to increased heart rate and cardiac output [11] and decreased blood viscosity [12] caused by anaemia. Although, recent emerging evidence suggests a strong link between anaemia of all categories and PPH [13, 14], most previous clinical studies investigating the connection between prenatal anaemia and PPH have reported conflicting results [8]. The 1997 case-control study conducted by Selo-Ojeme and Okonofua in Ile-Ife Nigeria did not identify anaemia as a significant risk factor for PPH [15] while the data from the 2017 international randomized trial on the effect of early tranexamic acid administration on mortality, hysterectomy, and other morbidities in women with post-partum haemorrhage (WOMAN) revealed a significantly higher risk of primary PPH in anaemic pregnant women enrolled from Nigeria [14, 16]. Therefore, more empirical research on this link and its magnitude may be necessary to inform decisions about maternal health policy given the high prevalence of prenatal anaemia and the importance of PPH for public health.

In this regard, we examined the association between maternal prenatal anaemia and primary PPH in a large cohort of women in five health institutions in Lagos, Southwest Nigeria. By evaluating the strength of this relationship, we are enhancing our comprehension of this urgent global health concern and laying the groundwork for implementing strategies to reduce the impact of PPH in resource-constrained settings, such as Nigeria.

## Participants and methods

### Study design and setting

In our prospective cohort analysis, we used data from the *“Predict-PPH”* study [17]. “*Predict-PPH*” is a recently conducted prospective cohort study of healthy pregnant women between the ages of 15 and 49 years, with gestation ranging from 28 to 36 weeks. The participants were recruited from the antenatal clinics of five healthcare institutions in Lagos, Nigeria from January to June 2023 [17]. These included three tertiary facilities– Lagos University Teaching Hospital (LUTH) in Idi-Araba, Federal Medical Center Ebute Meta (FMC-Eb) in Ebute Meta, and Lagos State University Teaching Hospital (LASUTH) in Ikeja; and two secondary facilities– 68 Nigerian Army Reference Hospital (68-NARHY) in Yaba, Lagos Island Maternity Hospital (LIMH) in Lagos Island. These hospitals are the foremost public health institutions in Lagos State, Southwest Nigeria, and they act mainly as referral centres for other government-owned and private hospitals in Lagos and its surrounding States. They all have established obstetrics and gynaecology departments with comprehensive maternity units that focus on providing care for pregnant women, childbirth, and postnatal care. The five facilities account for a cumulative annual delivery of 15,700 [17].

### Eligibility criteria

Participants in the primary study were consecutively consenting healthy pregnant women aged 15–49 years and at 28–36 weeks of gestation who attended the antenatal clinics of the five study sites [17]. In this current study, we analyzed the data of the *n* = 1189 women with a complete dataset from the primary study [17]. Excluded from the outcome analysis of the primary datasets were women who were unwilling to continue in the study or withdrew their consent at any time during the follow-up period, and those who presented for the first time after 36 weeks’ gestation or in labour. Further exclusion in this current study was the data of women who had intrauterine fetal death or died during the follow-up period. Several measures were instituted before and during the primary study [17] to standardize data collection across the study sites. These included the use of a standardized documented study manual for data collection, comprehensive training to all research staff including the study coordinators, site investigators, and research assistants on protocol adherence, data collection techniques, and ethical considerations., pilot testing of the study questionnaires and data collection procedures at each study site before full-scale implementation, inter-rater reliability checks to assess the consistency of data collection among the research assistants, and regular monitoring and supervision to oversee data collection activities, provide feedback to staff, and address any challenges or issues that arise.

### Extracted variables of interest

Variables extracted for analyses in the dataset included site and type of enrolment facility, and the women’s antepartum data such as gestational age at enrolment in weeks, age in years, pre-pregnancy or first-trimester body mass index (BMI) in kg/m^2^, number of previous childbirths, marital, educational and employment status, mode of conception (spontaneous or assisted), type of pregnancy (singleton or multiple), ultrasound diagnosis of uterine fibroids, antepartum bleeding in index pregnancy, Hb concentration at enrolment, measured in grams per deciliter (g/dL), was determined using the HemoCue® B-Hemoglobin system, postpartum blood loss in milliliters (mL) quantified directly using a blood collection V-drape and measured during the first 24 h of delivery. The HemoCue® system (HemoCue®, Ängelholm, Sweden) is a rapid and convenient point-of-care method used particularly in resource-limited settings. When used across diverse populations and clinical scenarios it produces comparable results as those obtained from laboratory-based reference methods, such as automated haematology analyzers or cyanmethemoglobin methods [18, 19]. The V-drape is a calibrated under-buttock drape that is folded out into a large sterile surface for delivery. A fluid pouch at the bottom of the sterile area holds more than 2500 mL of fluid and is marked at 50 mL intervals, thus allowing for precise measurement of postpartum blood loss. The collection pouch includes a flexible plastic filter to trap non-liquid materials and indicates when 500 mL postpartum blood loss has been collected [17]. When used in resource-limited settings, the drape could diagnose PPH at a rate four times that of the visual method of blood loss estimation [20].

### Variables of interest and operational definitions

As per the definition by the World Health Organization (WHO), anaemia is described as haemoglobin (Hb) concentration below 11 g/dL [21]. Anaemia in enrolled women in the study was further categorized based on the Hb concentration as mild anaemia (10 to < 11 g/dL), moderate anaemia (7 to < 10 g/dL), and severe anaemia (< 7 g/dL). The primary exposure variable was prenatal anaemia described as haemoglobin (Hb) concentration below 11 g/dL [21] at any time during enrollment. The primary study outcome was defined as the difference in the incidence of WHO-defined primary postpartum haemorrhage (PPH), described as postpartum blood loss of more than 500 mL within the first 24 h of delivery [22, 23], in women with and without prenatal anaemia. The secondary outcome was the differences in the median quantified postpartum blood across women with the different categories of anaemia.

### Sample size calculation

To investigate the effect of prenatal anaemia on the study outcome, WHO-defined PPH, we used an estimated weighted effect size of 0.5 for a two-sided test and a type I error rate of 5% to achieve a power of 80%, that is, Zα = 1.96 and Zβ = 0.84, adjusted for a 20% attrition rate to give a sample size of 498. Therefore, in the statistical analyses, we included all *n* = 1189 women with complete outcome data from the primary [17].

### Statistical analysis

Statistical analysis was conducted using IBM SPSS Statistics for Windows, Version 28.0 (IBM Corporation, Armonk, NY, USA). The Kolmogorov-Smirnov test with Lilliefors’ significance correction was employed to evaluate the normality of continuous variables. Subsequently, descriptive statistics were computed for the participants’ clinical and obstetric characteristics. Categorical variables were presented as frequencies and percentages, while continuous variables were depicted as mean (± standard deviation) for normally distributed data or median (interquartile range) for distributions exhibiting skewness. Univariable analyses of prenatal anaemia and other important variables selected a priori from the literature [24–31], that are potential risk factors of PPH were performed using Pearson’s Chi-square test. We assessed the multicollinearity across all the variables in the univariable analysis using variance inflation factors. We eliminated collinearity and adjusted for possible confounders in the association between maternal prenatal anaemia and PPH using a multivariable binary logistic regression model with the backward stepwise elimination approach. Akaike’s Information Criterion (AIC) was continuously computed, and the final model steps with the lowest AIC were chosen as the best-fit models. A stratified/interaction analysis was conducted to identify possible interaction effects in the model and subsequently, a sensitivity analysis was performed to eliminate these interaction effects to yield a more robust estimate of the odds ratio (OR) and confidence interval (CI). The Kruskal-Wallis test was used to compare the postpartum blood loss across women with different categories of anaemia. Associations in the study were regarded as significant if *P* < 0.05.

## Results

### Participants baseline information

**Fig. 1:**
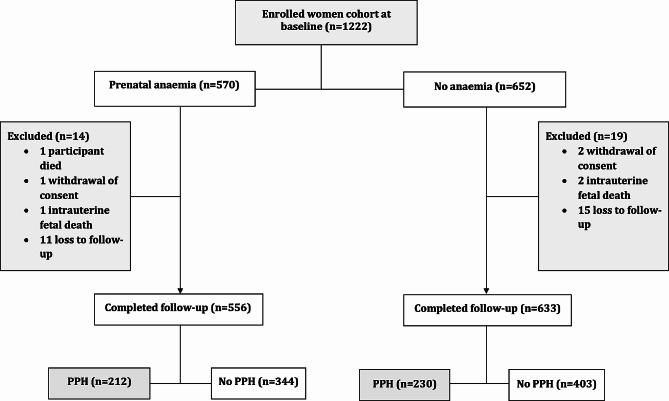
Participants study flow chart

In the primary study [17], *n* = 1222 women were enrolled over three months (January to March 2023) and followed up for five months (February to June 2023). Of these, one died before delivery, three withdrew their consent during the study for personal reasons, three experienced intrauterine fetal demise, and twenty-six were lost to follow-up. Therefore, of the enrolled women at baseline, 1189 (97.3%) had complete clinical data available for analysis after completion of follow-up and of these, 442 (37.2%) had WHO-defined PPH [Fig. 1]. There was no significant difference in the baseline characteristics between women who were excluded (*n* = 33) and those who were retained in the study (*n* = 1189) after enrollment.

Of the enrolled *n* = 1222 women at baseline, 570 (46.6%) were found to be anaemic based on their haemoglobin status. Among these anaemic women, 389 (68.2%) had mild anaemia, 175 (30.7%) had moderate anaemia and 6 (1.1%) had severe anaemia. The mean participants’ age at baseline was 30.7 ± 5.3 years, and the gestational age was 31.7 ± 2.5 weeks. There were statistically significant differences in the study sites (*P* < 0.001), type of study site facility (*p* < 0.001) and obesity (< 0.001) between the anaemic and non-anaemic participant groups. Table 1 displays the baseline characteristics of the women cohort stratified by their anaemic status.

**Table 1:** Baseline characteristics of participants stratified by their anaemia status (*n* = 1222)

Characteristics	Overall participants (%)	Anaemia (%)	No anaemia (%)	*P*-value
**Participants**	1222 (100.0)	570 (46.6)	652 (53.4)	
**Mean age (± SD) in years**	30.7 ± 5.3	30.3 ± 5.2	31.0 ± 5.4	0.941
**Mean gestation age at enrolment (± SD) in weeks**	31.7 ± 2.5	31.5 ± 2.5	31.9 ± 2.6	0.193
**Mean BMI (± SD) in kg/m** ^**2**^	27.9 ± 5.9	26.9 ± 5.5	28.8 ± 6.0	0.246
**Obesity**				
Yes (BMI ≥ 30 kg/m^2^)	374 (30.6)	142 (38.0)	232 (62.0)	< 0.001
No (BMI < 30 kg/m^2^)	848 (69.4)	428 (50.5)	420 (49.5)	
**Enrolment site**				
LUTH	148 (12.1)	67 (45.3)	81 (54.7)	< 0.001
LASUTH	161 (13.2)	64 (39.8)	97 (60.2)	
LIMH	522 (42.7)	271 (51.9)	251 (48.1)	
FMC-Eb	185 (15.1)	62 (33.5)	123 (66.5)	
68-NARHY	206 (16.9)	106 (51.5)	100 (48.5)	
**Level of enrolment facility**				
Secondary	728 (59.6)	377 (51.8)	351 (48.2)	< 0.001
Tertiary	494 (40.4)	193 (39.1)	301 (60.9)	
**Parity**				
Nullipara	482 (29.8)	231 (47.9)	251 (52.1)	0.632
Primipara	376 (70.2)	168 (44.7)	208 (55.3)	
Multipara	364 (70.2)	171 (47.0)	193 (53.0)	
**Marital status**				
Single	8 (0.7)	3 (37.5)	5 (62.5)	0.198
Married	1207 (98.8)	566 (46.9)	641 (53.1)	
Widowed	7 (0.6)	1 (14.3)	6 (85.7)	
**Educational status**				
No formal education	4 (0.3)	1 (25.0)	3 (75.0)	0.158
Primary education	18 (1.5)	5 (27.8)	13 (72.2)	
Secondary education	364 (29.8)	171 (47.0)	193 (53.0)	
Tertiary education	763 (62.4)	3666 (48.0)	397 (52.0)	
Postgraduate education	73 (6.0)	27 (37.0)	46 (63.0)	
**Employment status**				
Unemployed	96 (7.9)	46 (47.9)	50 (52.1)	0.100
Housewife	38 (3.1)	25 (65.8)	13 (34.2)	
Artisan	71 (5.8)	33 (46.5)	38 (53.5)	
Trading	583 (47.7)	253 (43.4)	330 (56.6)	
Civil servant	199 (16.3)	99 (49.7)	100 (50.3)	
Private professional	235 (19.2)	114 (48.5)	121 (51.5)	
**Mode of conception**				
Assisted	18 (1.5)	9 (50.0)	9 (50.0)	0.774
Spontaneous	1204 (98.5)	561 (46.6)	643 (53.4)	
**Type of pregnancy**				
Multiple	26 (2.1)	13 (50.0)	13 (50.0)	0.729
Singleton	1196 (97.9)	557 (46.6)	639 (53.4)	
**Presence of uterine fibroids**				
Yes	150 (12.3)	64 (42.7)	86 (57.3)	0.297
No	1072 (87.7)	506 (47.2)	566 (52.8)	
**Antepartum bleeding in the index pregnancy**				
Yes	106 (8.7)	55 (51.9)	51 (48.1)	0.258
No	1116 (98.3)	515 (46.1)	601 (53.9)	
**Previous hypertensive disorder in pregnancy**				
Yes	43 (3.5)	17 (39.5)	26 (60.5)	0.341
No	1179 (96.5)	553 (46.9)	626 (53.1)	
**Current hypertensive disorder in pregnancy**				
Yes	72 (5.9)	29 (40.3)	43 (59.7)	0.264
No	1150 (94.1)	541 (47.0)	609 (53.0)	
**Previous caesarean delivery**				
Yes	277 (22.7)	120 (43.3)	157 (56.7)	0.207
No	945 (77.3)	450 (47.6)	495 (52.4)	
**Previous PPH**				
Yes	27 (2.2)	9 (33.3)	18 (66.7)	0.161
No	1195 (97.8)	561 (46.9)	634 (53.1)	

Abbreviations: BMI, body mass index; FMC-Eb, Federal Medical Center Ebute-Meta; IQR, interquartile range; LASUTH, Lagos State University Teaching Hospital; LIMH, Lagos Island Maternity Hospital; LUTH, Lagos University Teaching Hospital; 68-NARHY, 68 Nigerian Army Reference Hospital Yaba; SD, standard deviation

### Effect of prenatal anaemia on the risk of PPH

In Table 2, after controlling for potential confounding factors such as level of enrolment facility, enrolment gestational age, women’s age, multiparity, obesity, mode of conception, type of pregnancy, any antepartum bleeding, uterine fibroids, previous hypertensive disorder in pregnancy, hypertensive disorder in the current pregnancy, previous caesarean delivery, previous postpartum haemorrhage, mode of delivery, and delivery gestational age in a multivariable model, maternal prenatal anaemia was found to be independently associated with PPH (adjusted odds ratio = 1.35, 95% confidence interval: 1.02–1.78). However, a stratified/interaction analysis revealed significant interaction effects of coexisting uterine fibroids (*P* = 0.010) and mode of delivery (*P* = 0.009) on the effects of prenatal anaemia on PPH (*P* < 0.001). Elimination of these interaction effects following a sensitivity analysis yielded a lack of statistically significant association between prenatal anaemia and PPH (adjusted odds ratio = 1.27, 95% confidence interval: 0.99–1.64).

**Table 2:** Univariable and multivariable analyses of anaemia as a potential risk factor for WHO-defined postpartum haemorrhage (*n* = 1189)

	Number of women with PPH	Crude OR (95% CI)	Adjusted OR (95% CI)
**Haemoglobin status**			
Anaemic	212/556 (38.1%)	1.08 (0.85–1.37)	1.35 (1.02–1.78)^#^
Non-anaemic	230/633 (36.3%)	1 (reference)	1 (reference)
**Level of enrolment facility**			
Secondary	252/717 (35.1%)	0.80 (0.63–1.02)	0.90 (0.67–1.19)
Tertiary	190/472 (40.3%)	1 (reference)	1 (reference)
**Enrolment gestational age**			
≥ 32 weeks	213/612 (34.8%)	0.81 (0.64–1.03)	0.80 (0.61–1.06)
< 32 weeks	229/577 (39.7%)	1 (reference)	1 (reference)
**Participants age**			
≥ 35 years	130/285 (45.6%)	1.59 (1.21–2.09)	0.82 (0.58–1.15)
< 35 years	312/904 (34.5%)	1 (reference)	1 (reference)
**Previous childbirths**			
≥ 2	163/355 (45.9%)	1.69 (1.31–2.18)	1.33 (0.98–1.81)
< 2	279/834 (33.5%)	1 (reference)	1 (reference)
**Obesity**			
Yes (BMI ≥ 30 kg/m^2^)	213/374 (57.0%)	3.39 (2.62–4.37)	3.32 (2.47–4.46)
No (BMI < 30 kg/m^2^)	229/815 (28.1%)	1 (reference)	1 (reference)
**Mode of conception**			
Assisted	11/18 (61.1%)	2.70 (1.04–7.01)	1.16 (0.39–3.43)
Spontaneous	431/1171 (36.8%)	1 (reference)	1 (reference)
**Type of pregnancy**			
Multiple	13/26 (50.0%)	1.71 (0.79–3.73)	0.99 (0.38–2.54)
Singleton	429/1163 (36.9%)	1 (reference)	1 (reference)
**Antepartum bleeding**			
Yes	64/106 (60.4%)	2.84 (1.89–4.28)	1.75 (1.08–2.84)
No	378/1083 (34.9%)	1 (reference)	1 (reference)
**Uterine fibroids**			
Yes	74/148 (50.0%)	1.83 (1.29–2.59)	2.20 (1.47–3.31)
No	368/1041 (35.4%)	1 (reference)	1 (reference)
**Previous hypertensive disorder in pregnancy**			
Yes	24/43 (55.8%)	2.20 (1.19–4.06)	0.95 (0.45–2.00)
No	418/1146 (36.5%)	1 (reference)	1 (reference)
**Current hypertensive disorder in pregnancy**			
Yes	38/71 (53.5%)	2.04 (1.26–3.30)	1.05 (0.58–1.89)
No	404/1118 (36.1%)	1 (reference)	1 (reference)
**Previous caesarean birth**			
Yes	177/271 (65.3%)	4.64 (3.48–6.19)	1.39 (0.94–2.06)
No	265/918 (28.9%)	1 (reference)	1 (reference)
**Previous postpartum haemorrhage**			
Yes	19/27 (70.4%)	4.15 (1.80–9.56)	2.59 (1.01–6.69)
No	423/1162 (36.4%)	1 (reference)	1 (reference)
**Mode of delivery**			
Caesarean birth	302/474 (63.7%)	7.21 (5.54–9.38)	5.72 (4.05–8.06)
Vaginal birth	140/715 (19.6%)	1 (reference)	1 (reference)
**Delivery gestational age**			
≥ 38 weeks	325/881 (36.9%)	0.95 (0.73–1.25)	1.18 (0.86–1.63)
< 38 weeks	117/308 (38.0%)	1 (reference)	1 (reference)

WHO-defined postpartum haemorrhage is defined as blood loss of at least 500 mL in the first 24 h after giving birth. The multivariable model controls for level of enrolment facility, enrolment gestational age, women’s age, multiparity, obesity, mode of conception, type of pregnancy, any antepartum bleeding, uterine fibroids, previous hypertensive disorder in pregnancy, hypertensive disorder in current pregnancy, previous caesarean delivery, previous postpartum haemorrhage, mode of delivery, and delivery gestational age

^#^ Elimination of interaction effects of coexisting uterine fibroids and mode of delivery yielded a non-significant association between prenatal anaemia and PPH (adjusted odds ratio = 1.27, 95% confidence interval: 0.99–1.64)

Abbreviations: BMI, body mass index; CI, confidence interval; OR, adjusted odds ratio; PPH, postpartum haemorrhage

### Postpartum blood loss across categories of anaemia

**Fig. 2:**
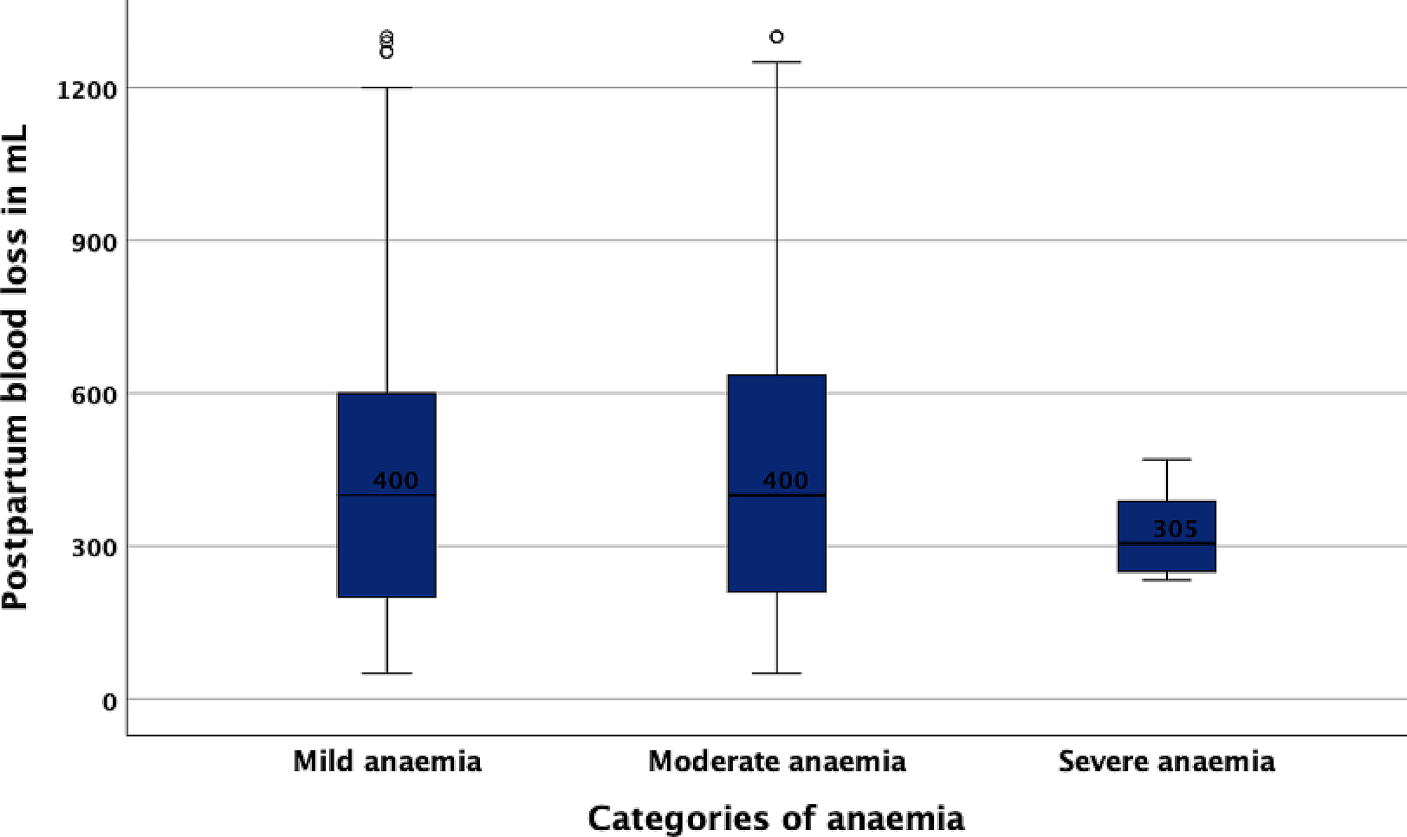
Simple boxplot showing the postpartum blood loss across different categories of anaemia in enrolled women with anaemia. The median blood loss was 400 mL for the 380 (68.3%) women with mild anaemia, 400 mL for the 171 (30.8%) women with moderate anaemia and 305 mL for the 5 (0·9%) women with severe anaemia (*P* = 0.131)

Analysis of the *n* = 556 enrolled women with anaemia revealed no statistically significant differences in the median postpartum blood loss across the different categories of anaemia (*P* = 0.131) [Fig. 2].

## Discussion

This prospective cohort analysis of the *Predict-PPH* study data revealed that almost half of the enrolled women presented with prenatal anaemia during the third trimester of pregnancy. Postpartum haemorrhage (PPH) was observed in more than a third of the enrolled participants and we also reported that prenatal anaemia was not significantly associated with PPH after adjusting for maternal covariates and elimination of variables of biological relevance to postpartum blood loss.

To our knowledge, this is the first prospective cohort study that assessed the association between prenatal anaemia and PPH in a single resource-limited country in Africa. The large sample size in the study allowed us to assess maternal anaemia, commonly seen in resource-constrained settings [32], using the standard WHO-defined haemoglobin cut-off value of 11 g/dL [21], and examine its effect on primary PPH. Prenatal haemoglobin concentration was measured using capillary haemoglobinometry (HemoCue® B-Hemoglobin system), which is accurate and almost comparable to the reference standard such as the automated haematology analyzer [19]. We assessed postpartum haemorrhage both quantitatively and objectively using the calibrated V-drape blood collection receptacle [20]. Furthermore, the robust study dataset allowed us to adjust for many potential confounding factors and eliminate any interaction effects on the observed association between prenatal anaemia and the risk of PPH.

The high prevalence of prenatal anaemia reported in this study in similarity to the magnitude of the condition previously reported among women of reproductive age in Western and Central Africa [2] further reinforced that anaemia remains frequent among pregnant women in low-income countries where haemoglobin screening is routinely recommended during pregnancy [32]. Also, the occurrence of PPH prevalence in more than a third of participants in the study regardless of their haemoglobin status is much higher than the global PPH prevalence of 10.8% [23]. This relatively higher PPH prevalence could be due to the variations in the criteria used to define PPH in this study and that of other previous studies [33, 34] which limit the comparability of our prevalence with that of these previous studies [33, 34]. It could also be due to our study settings which are predominantly referral public facilities, that are mostly patronised by women with high-risk pregnancies [35]. The higher PPH prevalence we recorded could also be the result of a more precise and objective measurement of postpartum blood loss due to the use of a novel calibrated blood collection V-drape [17]. Furthermore, the variation in the prevalence of our study from the recorded global prevalence may also be due to the paucity of effective medical interventions, and healthcare systems for controlling PPH in our settings such as the wide availability of essential treatments (heat-stable carbetocin, tranexamic acid, fresh frozen plasma, and platelet concentrates) [35, 36].

.

Despite variations in modelling assumptions and analytical approaches used in our study, the lack of a significant association between anaemia and PPH observed in our base model remained consistent. The identification and handling of interaction terms (anaemia and uterine fibroids; and anaemia and mode of delivery) further strengthened the observed associations, thus emphasizing the importance of considering interaction effects in logistic regression modelling. Several previous studies have documented an increased risk of PPH in women affected by anaemia. Shi et al. [13] in their secondary analysis of the data from China’s Hospital Quality Monitoring System showed that anaemia of all categories (mild, moderate, or severe) was associated with an increased risk of PPH compared with no anaemia. Furthermore, similar studies conducted in the sub-Saharan African countries of Tanzania [30], Senegal, Mali [37] and Nigeria [14, 16] found an increased risk of PPH with maternal anaemia. Some of the possible mechanisms that explain this finding include impaired oxygen transport due to anaemia that induces uterine atony [30, 38], impaired haemostasis and coagulation due to reduced circulating red blood cells [39], and increased blood flow from bleeding vessels due to increased heart rate and cardiac output [11] and decreased blood viscosity [12] caused by anaemia. However, contrary to the findings from these previous studies, our study is similar to another cohort study by Nair et al. in Assam, India [38] involving 1007 hospital-based births and a case-control study Selo-Ojeme and Okonofua in Ile-Ife, Nigeria [15] that reported no link between maternal anaemia and risk of PPH. Our study also reported no difference in the risk of PPH with increasing anaemia severity suggesting a lack of biological gradient or dose-response relationship between increasing anaemia severity and PPH risk [38], These conflicting findings further reinforced the need for future studies to explore the presence of a true causal relationship between PPH risk and anaemia of varying severity.

Our study has few limitations despite the large sample size. First, the study did not take into consideration anaemia that occurred at earlier gestational age or those that were corrected before delivery and the influence of this correction on the occurrence of PPH. Second, there are possible variations in the response to PPH management across the participating sites in the primary study that could affect our findings, but which were not controlled for in the data analysis. Third, the generalizability of the study findings is limited to clinical settings and among well-educated women, as the pregnant women participating in the study were specifically recruited from hospitals with over two-thirds of them having up to tertiary level of education. This has, therefore, excluded the majority of women in our setting who are mostly uneducated or partially educated and who predominantly undergo home-based deliveries. Finally, our findings could be an underestimated the magnitude of anaemia and its effect on PPH given that the primary study was conducted in women residing in the urban metropolitan areas of Lagos rather than in rural settings, where chronic, untreated anaemia is likely predominant and access to healthcare may be limited.

## Conclusions

Our study revealed that prenatal anaemia was not significantly associated with PPH. These findings challenge the previously held belief of a suspected link between maternal anaemia and PPH. This unique evidence contrary to most previous studies suggests that other factors beyond prenatal anaemia may contribute more significantly to the occurrence of PPH. This highlights the importance of comprehensive assessment and consideration of various maternal health factors in predicting and preventing this life-threatening obstetric complication. Furthermore, our findings underscore the need for further research to explore the multifaceted determinants of PPH. By elucidating the complex interplay of these factors influencing PPH, we can better tailor interventions and improve maternal health outcomes.

## Data Availability

The datasets used and/or analyzed during the current study are available from the corresponding author (KSO) upon reasonable request.
